# Secure Video Surveillance Framework in Smart City

**DOI:** 10.3390/s21134419

**Published:** 2021-06-28

**Authors:** Hao Li, Tianhao Xiezhang, Cheng Yang, Lianbing Deng, Peng Yi

**Affiliations:** 1 Post-Doctoral Research Center of Zhuhai Da Hengqin Science and Technology Development Co. Ltd., Zhuhai 519000, China; cuclihao@cuc.edu.cn (H.L.); denglb@dhqtech.com (L.D.); 2 State Key Laboratory of Media Convergence and Communication, Communication University of China, Beijing 100024, China; xiezth@cuc.edu.cn; 3 College of Information Engineering, Communication University of Chins, Beijing 100024, China; 4 Computer Science and Engineering, University of New South Wales, Sydney, NSW 2052, Australia; peng.yi@unsw.edu.au

**Keywords:** secure video surveillance, key management, decryption security, real-time performance

## Abstract

In the construction process of smart cities, more and more video surveillance systems have been deployed for traffic, office buildings, shopping malls, and families. Thus, the security of video surveillance systems has attracted more attention. At present, many researchers focus on how to select the region of interest (RoI) accurately and then realize privacy protection in videos by selective encryption. However, relatively few researchers focus on building a security framework by analyzing the security of a video surveillance system from the system and data life cycle. By analyzing the surveillance video protection and the attack surface of a video surveillance system in a smart city, we constructed a secure surveillance framework in this manuscript. In the secure framework, a secure video surveillance model is proposed, and a secure authentication protocol that can resist man-in-the-middle attacks (MITM) and replay attacks is implemented. For the management of the video encryption key, we introduced the Chinese remainder theorem (CRT) on the basis of group key management to provide an efficient and secure key update. In addition, we built a decryption suite based on transparent encryption to ensure the security of the decryption environment. The security analysis proved that our system can guarantee the forward and backward security of the key update. In the experiment environment, the average decryption speed of our system can reach 91.47 Mb/s, which can meet the real-time requirement of practical applications.

## 1. Introduction

With the continuous development of digital video cameras and network infrastructure, video surveillance is widely deployed in smart cities [1,2,3]. Moreover, the storage service for the public from cloud computing causes the cost of constructing video monitoring systems with different scales to become lower and lower. However, the accompanying security problems have become increasingly prominent [4,5].

Security and privacy issues exist widely in video surveillance systems deployed in smart cities. With the privacy leakage of Apple iCloud and the Verkada camera, the privacy of multimedia has attracted more and more attention. PatronuS [6] was proposed to find the region of interest (RoI) in videos quickly, and then the RoI was encrypted to achieve privacy protection in videos. Similarly, motion detection [7,8], skin detection [9], and compressed sensing [10] methods were proposed to find the proper RoIs for privacy protection. A lightweight encryption algorithm that was devised based on layered cellular automata (LCA) was proposed for fast RoI encryption [11].

However, privacy is a complex concept to define. It is even more difficult to define privacy areas in video, an unstructured file rich in various information [12]. It is not reasonable to simply regard the information of motion or skin as the RoI in practical application. For example, in traffic monitoring, privacy may be the license plate; privacy in hospitals may be electronic medical records (EMRs); and the privacy of station monitoring may be a passenger’s ID card, etc. It is a better choice to disturb the whole image with an acceptable encryption calculation. At the same time, the risk of privacy leakage may appear in the process of video collection, transmission, storage, and analysis [13]. Therefore, to fully protect privacy in multimedia, we need to realize data security during its whole life cycle in a media system.

The main challenge of data security faced by video surveillance systems is how to ensure the video integrity, confidentiality, and availability during the data life cycle. Sufficient encryption for multimedia is necessary. However, how to implement terminal authentication and key management securely is equally essential. In addition, many monitors exist in different locations of video surveillance systems, which need to obtain the suitable key and decrypt sensitive videos in specified areas. Hence, ensuring the monitor obtains the appropriate permission and builds a secure decryption environment is also meaningful. Monitors with excessive authorization may infringe upon other people’s privacy. Insufficient authorization of monitors may cause the video surveillance system to fail to work properly. For many small-scale scenes, such as homes and stores, a monitor is often replaced by a personal computer, which lacks the necessary security protection. It is obvious to ensure the security of the decryption environment. As a whole, in multi-level, multi-regional video surveillance systems for smart cities, the challenges mentioned above are unprecedented.

In our manuscript, we build a secure, convenient, and flexible video surveillance framework for use in a smart city. First, a trusted authority (TA) is built as a trust center of the city. Then, any terminal, whether a camera or a monitor, must be registered with the TA at the factory. When the terminal wants to access one video surveillance system, it should be authenticated to obtain the corresponding key legally. Specifically, the contributions of this manuscript include the following:

(1) A secure model is proposed to realize unified protection of video surveillance systems in smart cities. The model provides terminal authentication, key management, and secure video decryption for video surveillance systems with different scales, such as shops, families, and transportation.
(2) A secure authentication protocol is implemented for a secure video surveillance framework. Based on the Diffie–Hellman protocol, we add secret and timestamp elements that can effectively resist man-in-the-middle attacks (MITM) and replay attacks.
(3) Combined with the actual needs of smart city video surveillance, we build a hierarchical key management architecture named the Key Management Scheme Based on Normal Tree (KMSNT), which is based on the group key distribution and the Chinese remainder theorem (CRT). The TA manages the key of all video surveillance systems in the city. Group keys maintained by the TA are sent to video surveillance systems with different scales, such as families, shops, and buildings. When a group member changes, KMSNT updates the group key efficiently to ensure the data security.
(4) Considering that the decryption environment in a small-scale video surveillance system monitor may not be safe, we construct a video security decryption suite based on transparent encryption. It provides a secure environment for identity authentication, key acquisition, and data decryption of monitors. At the same time, it can prevent the terminal from illegally copying and modifying the video.

The remaining sections of this paper are organized as follows. The second part introduces related work. We prepare some relevant knowledge for our proposed scheme in Section 3. Section 4 proposes a secure video surveillance system model and describes the detailed algorithms for terminal authentication, key management, and secure video decryption. We analyze the security and real-time performance of the system in Section 5. Finally, Section 6 is the conclusion of our paper.

## 2. Related Work

We need to consider the security of video surveillance systems from two aspects. First, an adversary may access a video by illegal methods at a certain stage (video production, transmission, storage, etc.), leading to a privacy leakage. Second, an adversary may hijack the terminal to interfere with the normal work of the system. The related research can also be divided into two aspects: data protection and system security.

From the perspective of video data protection, digital video surveillance systems are mainly faced with three threats: confidentiality, integrity, and availability of the video. A secure video surveillance framework was proposed in [14] which used the AES symmetric encryption algorithm to encrypt the video stream, realized a symmetric key distribution through an RSA-based protocol, and employed hash-based message authentication message digest hashing (HMAC-MD5) to ensure the integrity of the video stream. Furthermore, hierarchical encryption and layered access control schemes were proposed in [15,16], in which an ordinary user can access the public video, while only authorized users are able to access the recovered private content. Generally speaking, encryption is still an important method to achieve privacy protection in video surveillance systems [17]. Naturally, it is increasingly urgent to build a secure and efficient key management scheme. Obviously, one-to-one key distribution protocols based on RSA or other asymmetric encryption algorithms are unsuitable for large-scale city monitoring systems because of the bottleneck in key management and distribution efficiency.

From the perspective of the attack surface of video surveillance systems, unauthorized video monitoring and stealing archived video footage are two common attack vectors used to attack video surveillance systems [18]. First of all, to ensure the safe running of a video surveillance system, any device connected to the system needs to be certified [19]. The dual-channel authentication approach using CCTV machine learning with radio frequency identification (RFID) was proposed [20]. The security of a video surveillance system depends on the secure storage of RFID and the accuracy of machine learning. However, an adversary attack proved that the machine learning algorithm can be misled [21]. Based on physically unclonable functions, authentication and key exchange protocols were proposed for Internet of Things (IoT) [22]. According to both [20] and [22], critical hardware is the foundation of security. Once the hardware information is leaked, the security of the system is no longer guaranteed. A role-based access control video surveillance mechanism was proposed in [23], which used a blockchain to guarantee the integrity of shared data and prevent indiscriminate access. However, a blockchain has the defects of heavy computing and a low efficiency. In practical applications, video surveillance systems with different scales need a set of secure, convenient, and flexible authentication and key distribution protocols to cooperate with video encryption algorithms of varying granularities and ultimately achieve the purpose of protecting the video data security. In addition, for small-scale scenes, such as homes and stores, the monitor is often a personal computer that lacks the necessary security protection. Therefore, how to ensure the security of the decryption environment in a monitor is also a very important problem.

## 3. Preliminaries

### 3.1. Group Key Distribution

**Figure 1:**
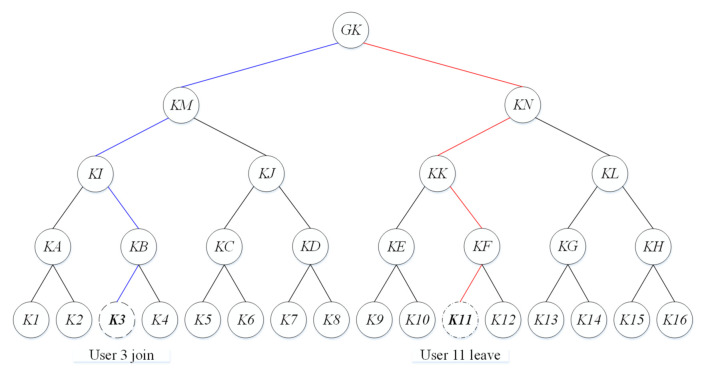
Group key update scheme-based LKH.

When a large number of terminals need to achieve encryption or decryption, the one-to-one key distribution protocol exposes the bottleneck of a low update efficiency and poor real-time performance. Group key management aims to achieve an efficient key update [24]. A group key update scheme-based logical key hierarchy (LKH) is shown in Figure 1. In the LKH structure, users or terminals are represented as leaf nodes.

**User join.** When user 3 joins the group, the group key *GK* and the internal keys (*KM, KI, KB*) belonging to the path from the root to the user 3 node are updated in the following steps to prevent the new user from obtaining the old keys.

(1) The new keys *GK′, KM′, KI′, KB′ * are encrypted by *K*3 and *K*4 and unicast to user 3 and user 4;
(2) *GK′, KM′, KI′* are encrypted by *KA* and multicast to users 1 and 2;
(3) *GK′, KM′* are encrypted by *KJ* and multicast to users 5 to 8;
(4) *GK′* is encrypted by *KN* and multicast to users 9 to 16.

**User leave.** When user 11 leaves the group, *GK, KN, KK, KF* need to be updated to ensure the forward security. The update steps are described below:

(1) The new keys *GK′, KN′, KK′, KF′* are encrypted by *K*12 and multicast to user 12;
(2) *GK′, KN′, KK′* are encrypted by *KE* and multicast to users 9 and 10;
(3) *GK′, KN′* are encrypted by *KL* and multicast to users 13 to 16;
(4) *GK′* is encrypted by *KM* and multicast to users 1 to 8.

The group key update scheme dramatically reduces the time consumption of the key update process. However, there is still room for optimization in the video surveillance system.

First, with the increase in users, the number of keys that users in the leaf node need to maintain increases heavily. When the number of users is *N*, the number of keys stored in the leaf node is $\log_{k}N+1$. *k* is the branch number of the tree. For example, when the user number is 16, the leaf node needs to maintain five keys.

Second, there are still several events of encryption and multicasting during the key update process. For example, when one user is evicted from the group, the new keys need to be distributed $\left(k-1\right)d$ times. Here, *k* is the number of branches, and *d* is the depth of the tree.

### 3.2. Chinese Remainder Theorem (CRT)

Assuming that *m*_1_, *m*_2_,…, *m_n_* are *n* pairwise relative prime numbers, then for any integer *a*_1_, *a*_2_,…, *a_n_*, the group of equations (Equation (1)) always has a unique integer solution modulo $N=m_{1}\times m_{2}\times \ldots \times m_{n}$.

(1) $$\left\{\begin{array}{c} \begin{array}{c} x\equiv a_{1}\left(\mathrm{mod}\;m_{1}\right)\\ x\equiv a_{2}\left(\mathrm{mod}\;m_{2}\right) \end{array}\\ \begin{array}{c} \ldots \\ x\equiv a_{n}\left(\mathrm{mod}\;m_{n}\right) \end{array} \end{array}\right.$$

If there are two integers *X* and *Y* that satisfy Equation (1), then $X\equiv Y\;\left(\mathrm{mod}\;N\right)$, where $N=\prod_{i=1}^{n}m_{i}$. Specifically, *x* can be calculated as follows:

(2) $$x\equiv \overset{n}{\underset{i=1}{\sum }}a_{i}\times \frac{N}{m_{i}}\times {\left[{\left(\frac{N}{m_{i}}\right)}^{-1}\right]}_{m_{i}}\left(\mathrm{mod}\;N\right)$$

In Equation (2), there is $\frac{N}{m_{i}}\times {\left[{\left(\frac{N}{m_{i}}\right)}^{-1}\right]}_{m_{i}}\equiv 1\left(\mathrm{mod}\;m_{i}\right)$. ${\left[{\left(\frac{N}{m_{i}}\right)}^{-1}\right]}_{m_{i}}$ is the inverse of $\frac{N}{m_{i}}$ modulo $m_{i}$.

Combined with the actual situation of video surveillance systems in smart cities, the CRT is used to improve the efficiency of the group key distribution.

### 3.3. Transparent Encryption in Video

Transparent encryption aims to provide a secure encryption and decryption environment, which protects the decryption in personal computers used as a monitor. Three transparent encryption algorithms for H.264/AVC and H.264/SVC compressed videos have been proposed [25]. All three schemes use encryption-after-encoding schemes. Taking advantage of the inter-layer prediction technique used in H.264/SVC (scalable video coding), a block-based encryption scheme (BBES) for encrypting H.264/SVC enhancement layers has been proposed [26]. Transparent encryption is used to encrypt a small amount of content adaptive binary arithmetic coding (CABAC) parameters to reduce the video quality and dampen any appetite for pirated copies [27]. In these studies, decryption is only briefly introduced because it has the same process as encryption. However, in the practical scenario, decryption has to face more security risks than encryption. One of the risks is that the decryption environment may not be secure. The solution proposed in [28] protects the decryption environment by a secure display path provided by ARM processors. Its security comes from a trusted root based on hardware, such as TrustZone [29]. For most personal computers, there is no trusted root module based on hardware. One scheme that revises the method of conditional access from one that depends on hardware CAMs within STBs to a software-based method of distribution was provided in [30]. The scheme is not based on hardware security. However, the decryption module is coupled in the application software, which is still not safe.

Therefore, we proposed a multimedia decryption middleware scheme based on transparent encryption. In the scheme, decryption is conducted by the transparent encryption driver provided by the operating system. The scheme can ensure that the plaintext only exists in memory and can only be used by authorized applications and prevent illegal disk writing and network transmission. However, only encryption is not enough for the authorized multimedia distribution. Authority control is also important. In our paper, key management and file decryption are integrated together as one middleware and provide a security service to the upper multimedia applications. This not only eliminates the coupling between the complex decryption process and the complex decoding process, reducing the difficulty and security requirements of the upper software development, but also improves the decryption efficiency and reduces the delay. The middleware completes a series of complex operations such as authentication, key acquisition, and decryption without the user’s awareness, which enhances the user experience.

## 4. Our Work

In this section, we propose a secure video surveillance system model including terminal registration, authentication, key management, and video encryption. Considering private protection in real life, a video surveillance based on cloud computing faces two security threats. (1) Traditional video streams are mainly transmitted and stored in a plaintext model, which leads to the risk of data leakage [31,32]. (2) Cloud-based video surveillance saves a lot of storage resources and provides a convenient and low-cost monitoring capability for small-scale scene applications such shops and families. However, shops and families often lack security awareness and protection skills, which makes web cameras and monitors (most of the time, the monitor is just a normal personal computer) vulnerable [33,34].

### 4.1. System Model

**Figure 2:**
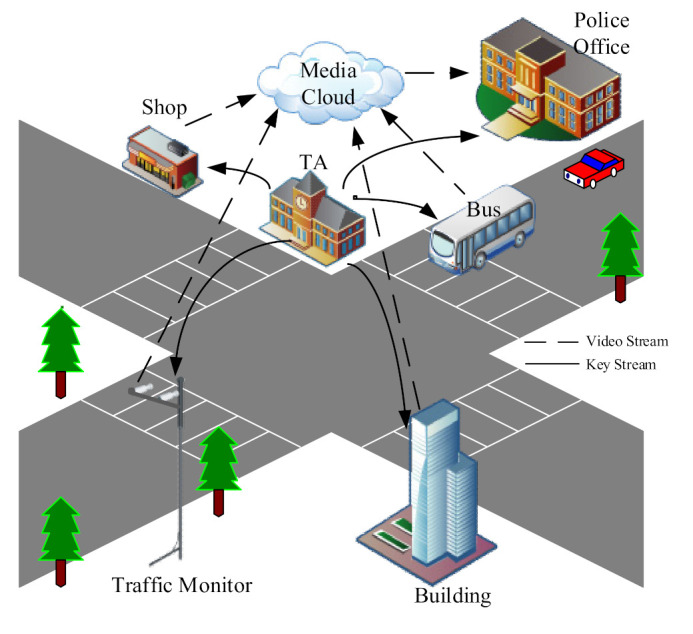
Secure video surveillance model in smart city.

In order to cover the above security risks, we built a typical smart city security video surveillance model (Figure 2). It is worth noting that we have not built this secure framework for a single video surveillance system, but for all video surveillance systems with different scales in smart cities. Taking a city as a unit, a unified secure video surveillance model was constructed. The model mainly consists of the following four subjects: trusted authority (TA), media cloud, monitor, and camera.

The **trusted authority (TA)** is responsible for the registration, authentication, and key management (key generation, distribution, storage, and destruction) of terminal devices, including monitors and cameras. The TA is trustworthy, and its structure can be central or hierarchical.

The **media cloud** is used to store and process monitoring videos. We believe the media cloud is curious, that is, the media cloud honestly follows the protocol execution, but curiosity propels it to speculate over and analyze the data and searchable index tree available at the server.

The **camera** is the basic video capture unit in the system. Multiple cameras can form a group to monitor a specific area, such as traffic, a shop, a family, and a building. In our system, any camera needs to be registered with the TA in its factory settings. Before the camera joins the video surveillance system, it should pass the security authentication. The legal camera captures and encrypts the video and then sends the encrypted video to the media cloud and local temporary storage server.

The **monitor** can view the videos captured by the cameras under its jurisdiction. In the actual scene, monitors and cameras have an obvious hierarchical structure. Simply, a monitor in a small shop can only view the video of the cameras belonging to the shop. A video surveillance system for an office building that has many independent companies has a more obvious hierarchy. One company’s monitor can only view the videos covering its office area but cannot view the videos beyond that area. However, the property company of the building has the right to view the surveillance video of the whole public area.

Figure 2 shows the secure video surveillance model used in a smart city. In the model, any terminal (monitor and camera) needs to register with the TA before it leaves the factory. The terminal sends the authentication request to the TA using its registration information when it wants to join the video surveillance system. After the authentication is finished, the TA classifies the terminal into the corresponding group for unified key management. For a legal camera, when the group key is received, the collected video is encrypted and sent to the media cloud and local temporary storage server. For a legal monitor, it has the right to access the video produced by the camera on its leaf nodes. Considering that a monitor may be a personal computer without any professional security protection in small-scale scenarios, we adopted transparent encryption to ensure the plaintext and decryption key do not exist in the computer memory. In particular, the model can provide a specific interface for the public police to resist crime.

**Figure 3:**
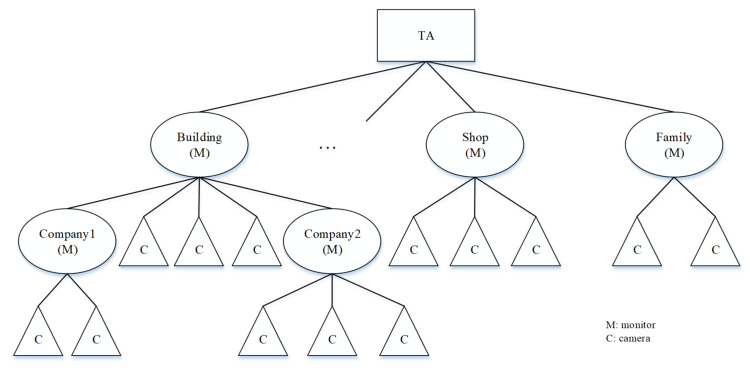
Hierarchical key management architecture.

Our model’s hierarchical key management architecture has three advantages for a secure video surveillance model in smart cities. (1) Cameras often work as a cluster, and hierarchical key management is more suitable for group key management for each cluster. (2) The hierarchical key management architecture has better visualization to represent the monitors and cameras in different regions and geographical locations. (3) The architecture can be easily extended, and the TA can be transformed from the central model to the distributed model, which is necessary for large-scale video surveillance in smart cities. Taking the central model architecture as an example (Figure 3), we define the TA to be on the root node, cameras to be on the leaf nodes, and monitors to be located on internal nodes.

### 4.2. Registration

Monitors and cameras need to register with the TA before they leave the factory. The registration process is initiated by the terminal *i* through the secure channel established between the TA and the factory. During this process, the TA records terminal information, including the manufacturer, registration time, device name, and type. Then, the TA assigns a unique public ID named $ID_{i}$ and a secret parameter $k_{i}$, where $ID_{i}$ is used as the identity of the terminal, and $k_{i}$ is used for future authentication.

### 4.3. Authentication

Before the device *i* joins the system, the TA and the device should be mutually authenticated. Firstly, the device *i* sends the authentication request message (containing $ID_{i}$, the parent node of *i* denoted as $ID_{parent_{i}}$, a random unique prime number *p*, and a random positive integer *g*) to the TA. Then, the TA starts the authentication phase and sends a message $auth_{i}$ to the terminal. The message contains the device ID $ID_{i}$, the unique identity of the TA $ID_{TA}$, the message authentication code ($MAC_{i}$), and the timestamp *T*. The message $auth_{i}$ is shown in Equation (3). The message authentication code $MAC_{i}$ is obtained by hashing the $ID_{i}$, terminal secret key $k_{i}$, $g^{a}\left(mod\;p\right)$, and *T*. In $MAC_{i}$, *a* is a positive integer randomly chosen by the TA:

(3) $$auth_{i}=ID_{i}∥ID_{TA}∥MAC_{i}∥g^{a}\left(mod\;p\right)∥T\;$$

(4) $$MAC_{i}=hash\left(ID_{i}∥ID_{parent_{i}}∥k_{i}∥g^{a}\left(mod\;p\right)∥T\right)\;$$

When the terminal *i* receives the message $auth_{i}$, $ID_{i}$, $ID_{TA}$, $MAC_{i}$, and *T* are separated from $auth_{i}$. Then, the terminal *i* calculates its own $MAC=hash\left(ID_{i}∥ID_{parent_{i}}∥k_{i}∥g^{a}\left(mod\;p\right)∥T\right)$, where *k_i_* is stored in the terminal *i* in the register processing. The *MAC* is compared with the received $MAC_{i}$. If the comparison is false, the authentication of the server fails. In addition, checking the timestamp *T* can resist replay attacks.

After passing the server authentication, the terminal *i* generates a random integer *b* and calculates $g^{b}\left(mod\;p\right)$. Then, the terminal *i* sends the response message $resp_{i}$ to the TA. $resp_{i}$ is shown in Equation (5), where $MAC_{i}^{\prime }=hash\left(ID_{i}∥k_{i}∥g^{ab}\left(mod\;p\right)∥\left(T+1\right)\right)$.

(5) $$resp_{i}=ID_{i}∥ID_{TA}∥MAC_{i}^{\prime }∥g^{b}\left(mod\;p\right)∥\left(T+1\right)$$

When the TA receives the response message $resp_{i}$ from $resp_{i}$ and the reserved information (*a*, *K_i_*, *ID_i_*, *ID_TA_*, and *T*), $MAC_{i}^{\prime }$ is easy to check. If it is equal, the authentication of the terminal *i* succeeds. After mutual authentication, the TA builds a secure channel with the terminal *i* and shares the secret $K_{i}=g^{ab}\left(mod\;p\right)$ for key management.

### 4.4. Key Management

For privacy protection in video surveillance, we propose the Key Management Scheme Based on Normal Tree (KMSNT). KMSNT realizes the group key distribution and provides secure methods for member join and leave to ensure backward and forward security. Aiming to solve slow and complex group key updates, we introduce the CRT to achieve simple and fast group key renewal.

There are three main functions in KMSNT: (1) group formation and group key distribution; (2) group key retrieval; (3) group member change. Additionally, the participants in KMSNT are the TA, monitor, and camera.

#### 4.4.1. Group Formation and Group Key Distribution

According to the devices’ authentication information, the TA maintains a large tree that denotes the grouping status of each monitor and its cameras. No matter the monitor or camera, the device *i* obtains the following data after authentication:$\;\langle ID_{i},g_{i},\;b_{i},p_{i},K_{i}=g_{i}^{a_{i}b_{i}}\left(mod\;p_{i}\right)\rangle$. We finish the group formation and group key distribution in this phase.

For each monitor *j*, the TA generates a group key $gk_{j}$ and a set of prime numbers $\left\{P_{i}|0<i\leq n\right\}$ randomly. The value *n* is the number of cameras in the group. Then, the TA prepares a message fragment *M_i_* for each camera. The message for the *i*th camera consists of the group key $gk_{j}$, the prime number $P_{i}$, a timestamp *T*, a message authentication code $MAC_{i}$, and the camera ID $ID_{i}$.

(6) $$MAC_{i}=hash\left(ID_{i}∥gk_{j}∥T∥P_{i}∥K_{i}\right)$$

(7) $$M_{i}=\left(ID_{i}∥gk_{j}∥P_{i}∥T∥MAC_{i}\right)$$

The final group key distribution message for the monitor *j* *C_j_* is encrypted by *K_i_* and sent to the group member.

(8) $$C_{j}=\left({\left\{ID_{i},\;E_{K_{i}}\left(M_{i}\right)\right\}}_{i=1}^{n}\right)$$

where *n* is the number of cameras belonging to the monitor $j.\;E_{k}\left(x\right)$ denotes that the information *x* is encrypted using the key *k*.

#### 4.4.2. Group Key Retrieval

When the camera *m* receives the ciphertext $C_{j}$ sent from the TA, the corresponding ciphertext fragment is intercepted according to the camera ID $ID_{m}$. Then, $M_{m}$ is released after the decryption. The camera validates the integrity of $MAC_{m}$ and checks the timestamp *T*. When the validation succeeds, the camera obtains the group key $gk_{j}$ and the prime number $P_{m}$ correctly.

Before the monitor *j′* accesses the encrypted data produced by its cameras, the monitor *j′* should be verified following the steps in Section 4.3. Then, the TA sends the group key message to the monitor *j′*. The group key is released following the same steps as those with the camera.

#### 4.4.3. Group Member Change

In order to ensure the security of the group key, the group key should be updated when the group members are changed. Member join and leave are two basic operations. Complex group changes, such as group merging and deletion, are made up of these two operations. In general, a group member change for video surveillance systems mainly occurs in the cameras because only the cameras are located on the leaf node.

**Member Join.** When a new camera wants to join the group, the TA should update the group key to ensure the new member cannot access the old key. This guarantees the backward security. Using the CRT, we realize a convenient key update.

Firstly, the TA calculates the value $N=\overset{n+1}{\underset{i=1}{\prod }}P_{i}$ where $P_{i}$ is the secret prime number of the camera *i* in the group. Additionally, *n* is the member number of the original group, and $P_{n+1}$ is the secret prime number of the new camera.

Secondly, the TA generates a new random key $gk_{j}^{\prime }$ for the group *j*. According to the CRT, we can obtain the following equations:

(9) $$\left\{\begin{array}{c} x\equiv \left(K_{1}⨁gk_{j}^{\prime }\right)\left(\mathrm{mod}\;P_{1}\right)\\ x\equiv \left(K_{2}⨁gk_{j}^{\prime }\right)\left(\mathrm{mod}\;P_{2}\right)\\ \ldots \\ x\equiv \left(K_{i}⨁gk_{j}^{\prime }\right)\left(\mathrm{mod}\;P_{i}\right)\\ \ldots \\ x\equiv \left(K_{n}⨁gk_{j}^{\prime }\right)\left(\mathrm{mod}\;P_{n}\right)\\ x\equiv \left(K_{n+1}⨁gk_{j}^{\prime }\right)\left(\mathrm{mod}\;P_{n+1}\right) \end{array}\right.$$

$K_{i}=g_{i}^{a_{i}b_{i}}\left(mod\;p_{i}\right)$ is the secret shared between the TA and the camera *i*. The value *P_i_* is the secret prime number produced by the authentication process. Since *P*_1_, *P*_2_, …, *P_n_*, *P*_*n*+1_ are a set of prime numbers selected randomly, they are pairwise relative prime integers and $K_{i}⨁gk_{j}^{\prime }\in \mathbb{Z}\;\left(i\in \left[1,n+1\right]\right)$. Therefore, the equations group has a unique solution modulo *N*.

(10) $$x\equiv \overset{n+1}{\underset{i=1}{\sum }}\left(K_{i}⨁gk_{j}^{\prime }\right)\times \frac{N}{P_{i}}\times {\left[{\left(\frac{N}{P_{i}}\right)}^{-1}\right]}_{P_{i}}\left(\mathrm{mod}\;N\right)$$

For each *i*, there is $\frac{N}{P_{i}}\times {\left[{\left(\frac{N}{P_{i}}\right)}^{-1}\right]}_{P_{i}}\equiv 1\left(\mathrm{mod}\;P_{i}\right)$. ${\left[{\left(\frac{N}{P_{i}}\right)}^{-1}\right]}_{P_{i}}$ is the inverse of $\frac{N}{P_{i}}$ modulo $P_{i}$. Then, *x* is sent to the whole new group members. When the *i*th camera receives *x*, the new group key $gk_{j}^{\prime }$ is renewed by the following equation:

(11) $$gk_{j}^{\prime }=\left(x\;mod\;P_{i}\right)⨁g_{i}^{a_{i}b_{i}}$$

**Member Leave.** When the old member leaves the current group, the TA should update the group key to maintain the forward security. The leaving member can no longer access the group key. For the traditional group key distribution scheme, the group key update finishes after $\left(k-1\right)d$ rounds of information exchange. The value *k* is the number of branches of the tree, and *d* is the depth of the tree. However, with the CRT, we only need one round of message exchange.

The steps of the group key update in member leave are similar to those in the member join process. To transmit the new group key $gk_{j}^{\prime }$ secretly, the reserved member in the group can make up the following equations:

(12) $$\left\{\begin{array}{c} \begin{array}{c} x\equiv \left(K_{1}⨁gk_{j}^{\prime }\right)\left(\mathrm{mod}\;P_{1}\right)\\ x\equiv \left(K_{2}⨁gk_{j}^{\prime }\right)\left(\mathrm{mod}\;P_{2}\right)\\ \ldots \end{array}\\ \begin{array}{c} x\equiv \left(K_{i}⨁gk_{j}^{\prime }\right)\left(\mathrm{mod}\;P_{2}\right)\\ \ldots \\ x\equiv \left(K_{n-1}⨁gk_{j}^{\prime }\right)\left(\mathrm{mod}\;P_{n-1}\right) \end{array} \end{array}\right.$$

Then, *x* is sent to the reserved group members. When the *i*th camera receives *x*, the new group key $gk_{j}^{\prime }$ is built by the following equation:

(13) $$gk_{j}^{\prime }=\left(x\;mod\;P_{i}\right)⨁g_{i}^{a_{i}b_{i}}$$

### 4.5. Secure Video Decryption

Video encryption has been fully discussed in many papers. We do not provide any new video encryption algorithm in this manuscript. However, we attempt to solve a new problem which is exposed in video surveillance systems. In some realistic video surveillance scenes, such as shops and families, the monitor is often a personal computer without any professional protection. This leads to the decryption environment of the monitor being complex and vulnerable. Based on transparent encryption, we designed a secure decryption scheme to resist vulnerable environments. The proposed scheme for monitors consists of a user module and a kernel module. The former is used for monitor authority control to communicate with the TA to obtain the decryption key. The kernel module finishes the video integrity validation and executes secure decryption to protect the plaintext from modification.

#### 4.5.1. Authority Control in User Module

Authority control is realized for monitors to obtain the decryption key legally. We assume that the monitor has registered in the TA earlier. Firstly, the monitor and TA verify each other according to the steps described in Section 4.3. If the validation fails, the video decryption process is finished. The monitor with a failed verification cannot access the encrypted video. Otherwise, the monitor requests the decryption key from the TA following the steps in the Group Key Retrieval function (Section 4.4).

The monitor *m* sends $ID_{m}$ to the TA and begins the decryption key request process. The TA receives the request and prepares a message $M_{m}$ for the monitor. The message consists of the corresponding group key $gk_{m}$, the prime number *P_m_*, a timestamp *T*, a message authentication code $MAC_{m}$, and $ID_{m}$.

(14) $$MAC_{m}=hash\left(ID_{m}∥gk_{m}∥T∥P_{m}∥K_{m}\right)$$

(15) $$M_{m}=\left(ID_{m}∥gk_{m}∥P_{m}∥T∥MAC_{m}\right)$$

The final group key distribution message $C_{m}$ for the monitor *m* is encrypted by $K_{m}$ and sent to the monitor *m*.

(16) $$C_{m}=\left\{ID_{m},\;E_{K_{m}}\left(M_{m}\right)\right\}\;$$

For the monitor *m* and TA, $K_{m}$ is the shared secret produced after the authentication process. Then, the monitor can easily decrypt the group key $gk_{m}$ as the decryption key from the group key distribution message $C_{m}$. All operations take place in the user module of transparent encryption to ensure that the sensitive information including $K_{m}$ and $gk_{m}$ is not leaked.

#### 4.5.2. Secure Decryption in Kernel Module

**Figure 4:**
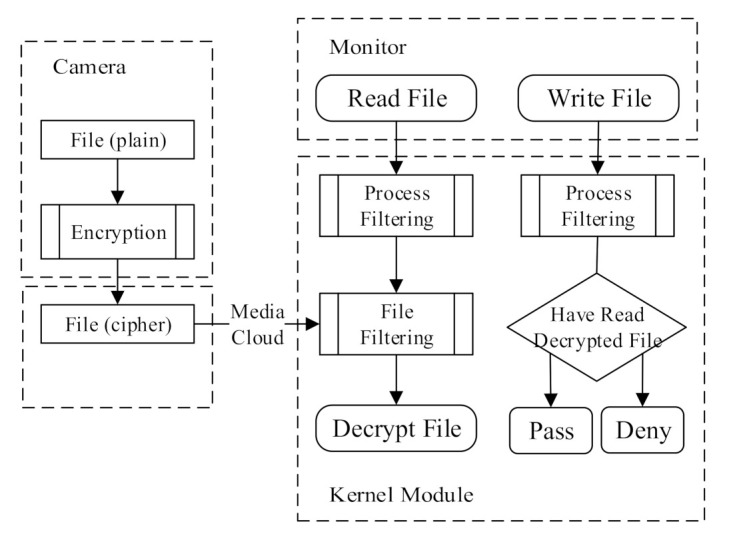
Encryption and decryption process.

When the monitor obtains the decryption key $gk_{m}$ correctly, the ciphertext will be decrypted using $gk_{m}$ in the kernel module of transparent encryption. This process is depicted in Figure 4.

We briefly describe the video encryption process firstly. For each encrypted multimedia segment, the encryption information shown in Table 1 is attached to the file header. Assuming that the plaintext *fData* is input into the encryptor, and the group key *fKey* has been prepared well, encryption follows the steps below:

(1) Encrypt *pData* with *fKey* and obtain the ciphertext *cData* = *fKey(pData)*;
(2) Generate the digest of *cData* represented by *digest* and put *digest* in the header of *cData*;
(3) Set *encFlag* to *ENC* in the prefix, which means the following data are encrypted;
(4) Pad the file prefix up to a multiple of 32 bytes;
(5) Count the number of padding data *PSize*. Then, the output is

File_Cipher=(encFlag, PSize, Digest, Padding, cData)

For symmetric encryption, decryption is the inverse process of encryption. However, in practical scenarios, decryption has to face more security risks than encryption. Specifically, (1) the environment for decryption may not be secure enough; and (2) the decrypted information may be abused or forged. In our scheme, we use two filters in the kernel module, named the process filter and the file filter, to ensure the security of the decryption environment and prevent the plaintext from being abused.

The process filter aims to prevent the plaintext from being leaked out by malicious applications or processes. During the filtering process, the validity of the application in the receiver side will be checked. The MD5 value of the application will be compared with the trusted application list. When the application comparison result is true, we set *Flag* = *True*. At the same time, the process filter uses writing blocking to reject the writing operation from any application or process.

After the process filter, file filtering is used to verify the integrity of the encrypted files and decrypt the ciphertext. If the encryption flag in the file header is true, the file integrity will be verified. File filtering has two steps, pre-operation and post-operation, as shown in Table 2. The cipher prefix (including *digest* and *encFlag*) preset in the file prefix will be read out, and *Flag* is checked in pre-operation. In post-operation, a new generated *digest*1 will be compared with *digest* to check whether the file data are identical to the original version before the decryption key request is sent out. Then, the ciphertext *cData* will be decrypted.

As the core function, the decryption process is realized based on a Minifilter. Integrity is inspected using the message digest algorithm simultaneously while accessing a file. In addition, sensitive applications and processes are kept from writing operations to eliminate data leakage and modification.

## 5. System Analysis

In this section, we analyze the security and the real-time performance of the proposed video surveillance scheme. In the security analysis part, we mainly focus on the forward/backward security of the group key management scheme and the monitor environment security during the video decryption process. For the real-time performance, we believe that the requirement of real time in video surveillance systems is in monitor decryption. There are two reasons. First, when the video surveillance system works smoothly, it is not common for a camera to join or leave frequently. Second, the camera that captures and encrypts the video can be specified and customized to meet the real-time performance. However, the monitor may be a personal computer with poor properties or a dedicated server with a strong calculation capacity. Therefore, it is more meaningful to discuss the real-time performance of decryption in monitors. In monitor decryption real-time performance analysis, the experiments were implemented by PHP and C++ and run on a computer (Windows 7 32-bit OS, 4*3.2 GHz CPU, 12 GB memory, and 16 MB + 7200 r/min HDD). All tests were carried out under the Gigabit LAN.

### 5.1. Security Analysis

In this section, we discuss the forward and backward security of the group key management scheme and the security of the monitor decryption environment.

#### 5.1.1. Forward Security

When any leaf node of the tree built in our key management scheme is evicted from the group, the group key update algorithm must guarantee forward security to prevent the evicted node from reading the new message. As shown in Section 4.4, the new group key is renewed by the equation $gk_{i}^{\prime }=\left(x\;mod\;P_{i}\right)⨁g_{i}^{a_{i}b_{i}}$, where *i* belongs to the remaining members in the group. The new group key is randomly generated by the TA and has no relation with the old key. For the evicted member, it is necessary to guess the secret parameters *P_i_* and $g_{i}^{a_{i}b_{i}}$ that belong to one of the participating members. However, $g_{i}^{a_{i}b_{i}}$ is shared only between the TA and the *i*th camera, and *P_i_* is also the secret prime number only known by the TA and the *i*th camera. Therefore, a brute force attack is the only way for the evicted member to obtain the correct group key. Hence, forward security is maintained.

#### 5.1.2. Backward Security

Similar to forward security, the secure anchor is on the security of the secret $g_{i}^{a_{i}b_{i}}$. As we know from Section 4.3, the $g_{i}^{a_{i}b_{i}}$ is shared between the TA and the *i*th camera. The secrecy of $g_{i}^{a_{i}b_{i}}$ is guaranteed by using the computational Diffie–Hellman assumption (CDH). To resist MITM and replay attacks, we add a message authentication code ($MAC_{i}$) and timestamp T into the CDH process. In $MAC_{i}$, *k_i_* is only shared between the TA and the *i*th terminal, meaning the MITM attack cannot work. The timestamp T is obtained when the authentication really occurs, and both the TA and terminal will verify the validity of T. The invalid T will cause an authentication failure. Then, the replay attack is resisted.

#### 5.1.3. Decryption Security

From the perspective of confidentiality, sensitive information includes multimedia data and secret keys. The main risk of them is that they may be stolen through devious ways. In a monitor, a sensitive video can be accessed only when the decryption is executed with a correct key. Additionally, after decryption, a Minifilter ensures that decrypted data are only obtainable for verified trusted applications until they are destroyed and will not be leaked out by disk writing or network transmission. The secret key is stored in the kernel memory and insulated from unauthorized access until it is destroyed, meaning the security of the secret key in the monitor can be maintained.

From the perspective of integrity, multimedia may be tampered or damaged. However, tampering definitely changes the digest of the file. When accessing a forged file, file filtering usually fails because the file cannot pass the integrity verification. Even if the digest on the file prefix can be replaced by that of the forged file, it is impossible to obtain a correct key that was indexed by the original file digest. Therefore, tampering will be detected immediately. Damage makes encrypted multimedia unusable. However, authorized users can retrieve an original copy from the cloud at any time. Unauthorized users cannot obtain any useful information from the damaged file.

### 5.2. Real-Time Analysis

#### 5.2.1. Secure Decryption Speed

As mentioned before, we focus on the decryption speed in the monitor. When the monitor obtains the decryption key, the next step is to use the key to decrypt the encrypted multimedia. In the decryption process, the validity of the application and the integrity of multimedia will be verified firstly. In the decryption test, we consider the efficiency of the decryption, that is, the decryption speed. The validity of the application happens before decryption, and it needs to communicate with the server. In the actual implementation, we submit the MD5 value of the application together with the key request to the server. That ensures all operations in filters are local.

**Figure 5:**
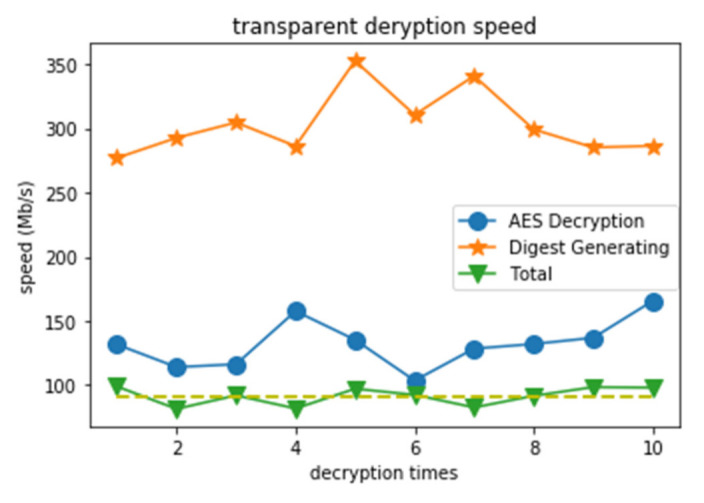
Speed of transparent decryption.

Therefore, there are two factors that affect the efficiency of the transparent decryption, the AES decryption speed and the digest generation speed. In this experiment, we used 128-bit ECB mode AES for encryption, and 256-bit MD5 for digest generation. The result is shown in Figure 5. In the total 30 tests, the first 10 are the results of only performing AES decryption; the next 10 are the result of only performing digest generation; the last 10 are the performance of the whole transparent decryption. As we can see from Figure 5, the AES decryption speed is about 132.16 Mb/s, and the digest generation speed is about 2.3 times the decryption speed, at about 303.57 Mb/s. Generally, the total transparent decryption speed is about 91.47 Mb/s.

#### 5.2.2. Comparison

The efficiency of multimedia encryption and decryption is closely related to the encryption method and encryption algorithm. In our demo, we adopt full encryption for multimedia, including videos. The decryption speeds for different types of multimedia using different encryption algorithms are shown in Table 3. Compared with the image encryption in [35,36,37] and the video encryption in [38], our scheme provides a much faster decryption speed, reaching 91.47 Mb/s. This indicates that our scheme can be applied in the decryption environment of the reference schemes and provide security and a real-time decryption service. In addition, if the decryption algorithm changes from full encryption to selective encryption, the demo can realize a better real-time performance.

## 6. Conclusions

More and more video surveillance systems have been deployed in the smart cities now. The security of video surveillance faces serious challenges, especially video data. The main challenge of data security faced by video surveillance systems is how to ensure the video integrity, confidentiality, and availability. The key to solving the problem is to build an appropriate scheme for device authentication, key management, and data encryption/decryption to prevent criminals from attacking the system on different attack surfaces. In this manuscript, we built a secure video surveillance model which includes four participants: trusted authority (TA), media cloud, monitor, and camera. Then, terminal registration, mutual authentication, and a group key management method named KMSNT were implemented. By adding secrets and timestamps into the Diffie–Hellman protocol, the proposed mutual authentication protocol can resist MITM attacks and replay attacks. Security analysis proved KMSNT can guarantee the forward and backward security of the key update. Considering the decryption environment in monitors of small-scale video surveillance systems may not be safe, we constructed a video security decryption suite based on transparent encryption. The suite provides a secure environment for identity authentication, key acquisition, and data decryption in the monitors. At the same time, it can also prevent the terminal from illegally copying and modifying the video. The video security decryption scheme has a fast decryption speed which can reach 91.47 Mb/s on average.

**Future work**. We are considering building a more complex tree to achieve more practical key management requirements in video surveillance systems, such as regional division and cross-level access. We will also try to optimize the video encryption scheme to improve the encryption efficiency and reduce the encryption consumption in the cameras.

## Figures and Tables

**Table 1:** Prefix format.

Name	Size (Byte)	Meaning
encFlag	4	If the file is encrypted
PSize	4	Size of file prefix
Digest	32	Digest of cipher data
Padding	-	Prefix padding

**Table 2:** File filtering process.

Pseudocode 1: Pre-Operation	Pseudocode 2: Post-Operation
**Input:** application comparison result Flag, file path fpath. **Output:** preset digest of cipher data digest. 1 … 2 var prefix=read_file_header(fpath); 3 **if** prefix=′ENC′ AND Flag=True **then** 4 var digest=prefix.digest; 5 var psize=prefix.psize; 6 file_read_start_position +=psize; 7 **End** 8 …	**Input:** preset digest of cipher data digest, read the file index findex and the data fdata. **Output:** read file data fdata. 1 … 2 var cData=fdata; 3 var digest1=generate_digest(cData); 4 **if** digest1=digest **then** 5 var key=get_fileKey(findex); 6 var pData=decrypt(cData,key); 7 fdata=pData; 8 **End** 9 …

**Table 3:** Decryption speed comparison.

Schemes	[35]	[36]	[37]	[38]			Proposed Scheme
Multimedia type	Image			Video			All
Encryption algorithm	Chaos-based			AES	RC6	DES	All
Speed (Mb/s)	0.45	1.85	1.87	17.89	8.94	3.59	**91.47**

## Data Availability

Not applicable.
